# Disseminated Peritoneal Leiomyomatosis: An Unusual Incidental Finding During Cesarean Delivery—A Case Report

**DOI:** 10.1002/ccr3.72450

**Published:** 2026-04-03

**Authors:** Seblewengel Maru Wubalem, Shemsu Abraham Hussien, Endalk Shambel Ayalew, Kidest Melkamu Gebeyehu

**Affiliations:** ^1^ Department of Pathology Wachemo University Hossana Ethiopia; ^2^ Department of Obstetrics and Gynecology Wachemo University Hossana Ethiopia; ^3^ Department of Pathology St. Peter Specialized Hospital Addis Ababa Ethiopia

**Keywords:** disseminated peritoneal leiomyomatosis, histopathology, peritoneal tuberculosis, pregnancy

## Abstract

Disseminated peritoneal leiomyomatosis (DPL) is a rare benign condition featuring smooth muscle nodules. In a 27‐year‐old patient, intra‐abdominal lesions mimicking tuberculosis were discovered incidentally during a cesarean section. Biopsy confirmed DPL, highlighting its importance in the differential diagnosis of disseminated abdominal masses to avoid misdiagnosing malignancy or infectious diseases.

## 1 Introduction

Disseminated peritoneal leiomyomatosis (DPL) is a rare benign tumor that is characterized by numerous small smooth muscle nodules disseminated in the abdominal and pelvic cavity. It is seen in reproductive‐age women and rarely in postmenopausal women. There are fewer than 200 cases reported so far [1, 2, 3, 4]. It was first described by Wilson and Peale in 1952 [5]. DPL can be challenging to diagnose since it resembles peritoneal carcinomatosis or other disseminated intra‐abdominal malignancies [6, 7].

We present a case of a 27‐year‐old G3P1 (alive, spontaneous vaginal delivery) A1 (spontaneous at 12th week of gestation) Ethiopian mother who was diagnosed with DPL as an incidental finding during cesarean section (CS) delivery for an indication of cord prolapse. Her clinical diagnosis was intra‐abdominal disseminated tuberculosis (TB). However, the histologic examination revealed a benign smooth muscle tumor, DPL.

## 2 Case History / Examination

A 27‐year‐old G3P1A1 Ethiopian mother, at a gestational age of 41^+4^ weeks, presents to the obstetrics emergency department with a gush of fluid per vagina of 1‐h duration with associated pushing down pain of 3 h duration. Otherwise, she has no history of vaginal bleeding, fever, previous gynecologic surgery, oral contraceptive use, or any known chronic illness.

On physical examination, the vital signs were stable. Gravid uterus with cephalic presentation of the fetus and uterine contractions appreciated on abdominal examination. There was a palpable umbilical cord in the vaginal canal that was picked up by vaginal examination.

## 3 Differential Diagnosis/Investigations/Treatment

**FIGURE 1:**
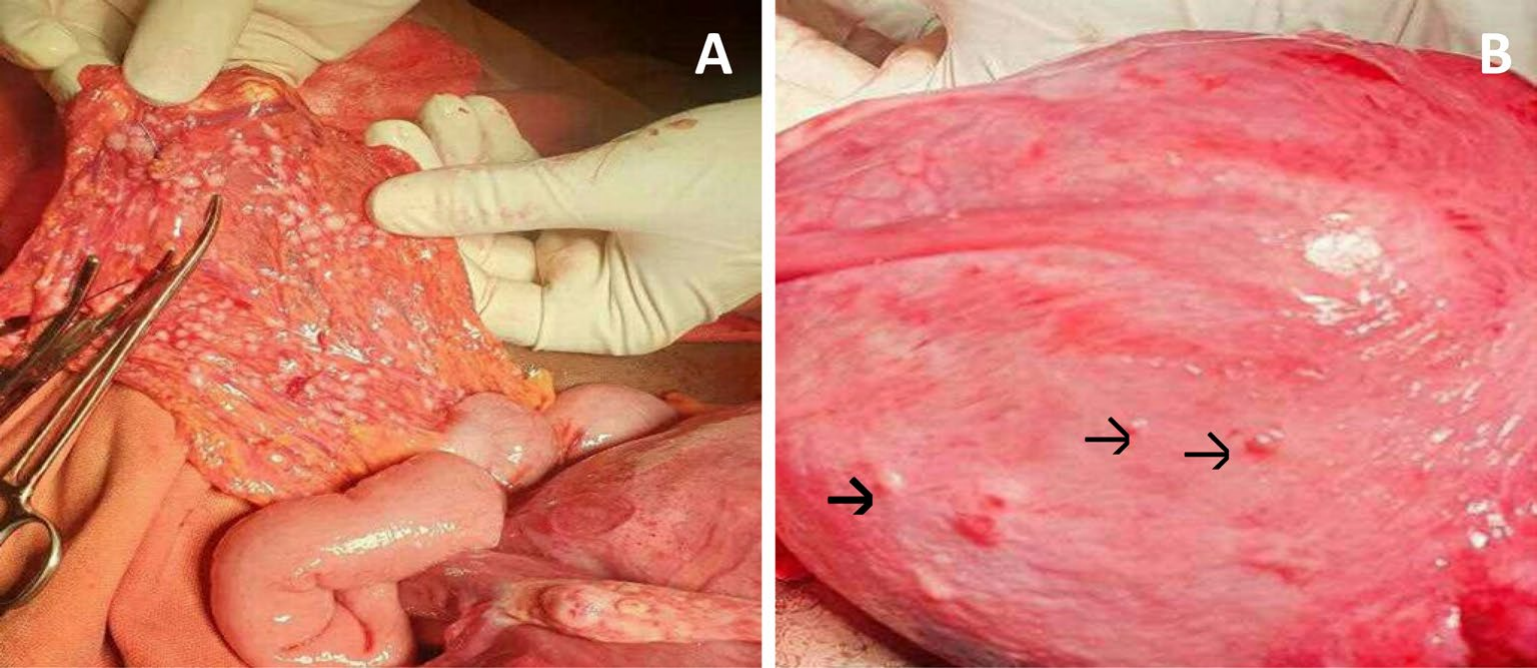
Intraoperative pictures: (A) Omental nodules attached to ileum. (B) Nodules over the uterine surface (arrows).

On investigations, complete blood count was within normal range. The routine obstetric ultrasounds did not detect any abnormality. Therefore, she was taken to the operating theater to do an emergency CS for an indication of cord prolapse. Multiple disseminated white nodular lesions were noted incidentally over the omentum and ileum while exploring the peritoneum after the delivery of a 3.5 kg live male fetus. Similar tiny nodules were observed on the serosal surface of the uterus (Figure 1). All of the nodules were resected except the small nodules over the uterine serosa. The lesions were assumed clinically to be disseminated TB, and a biopsy was sent to the pathology department for histologic examination.

**FIGURE 2:**
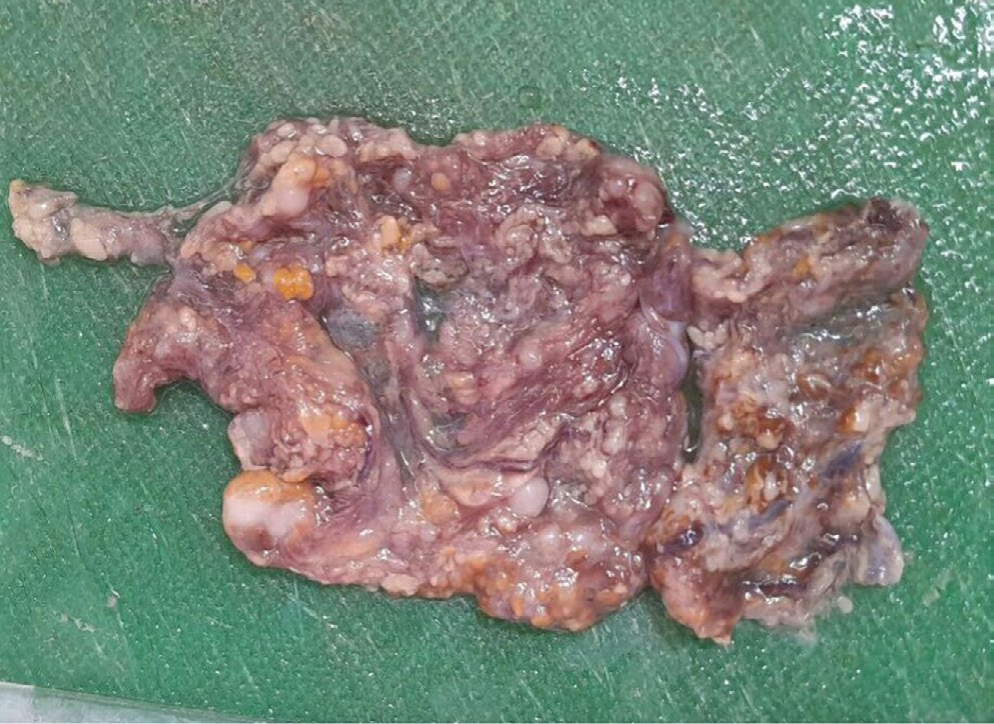
Gross picture: membranous fibrofatty tissue with multiple gray‐white solid nodules.

**FIGURE 3:**
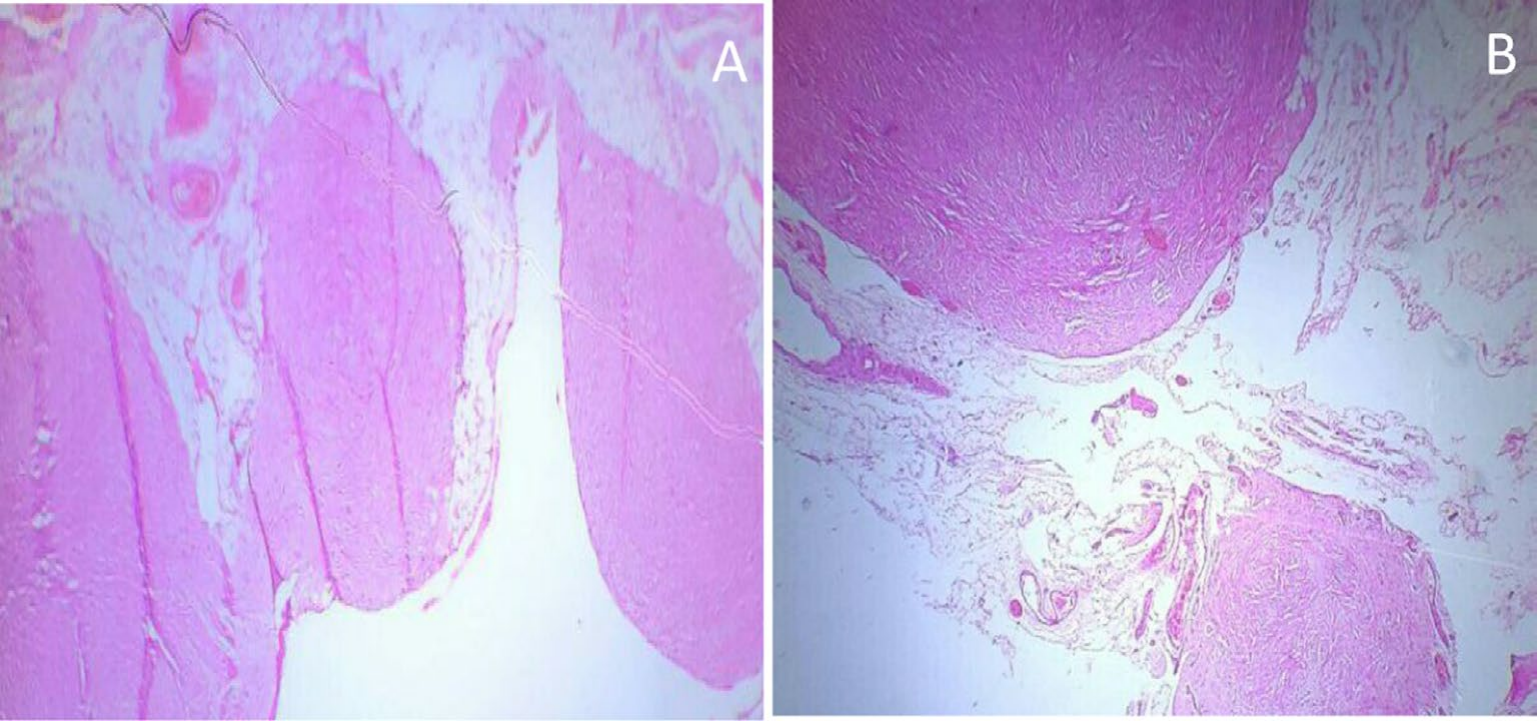
Histopathology pictures show multiple well‐circumscribed nodules of DPL (hematoxylin and eosin, × 40).

**FIGURE 4:**
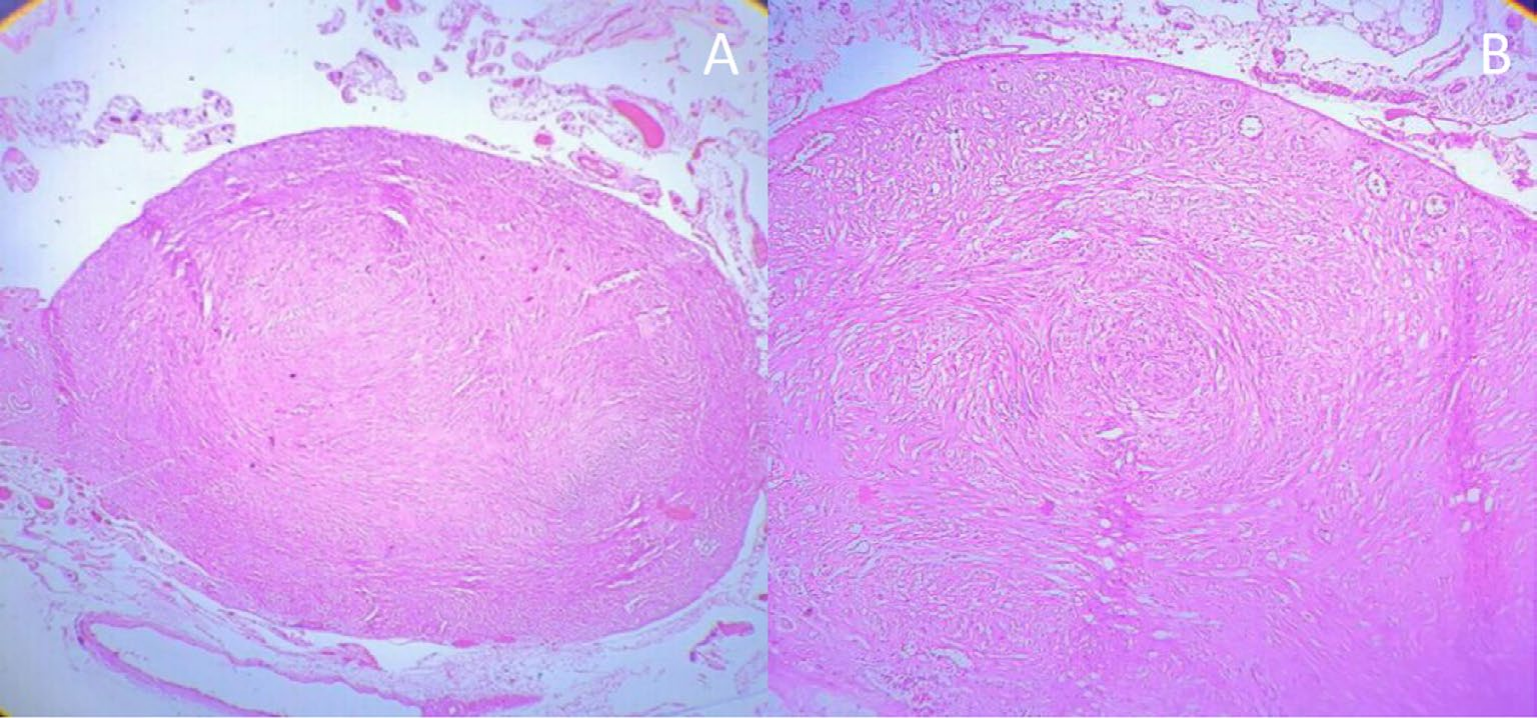
A and B: Histopathology picture showing well‐circumscribed nodules forming whorls and intersecting fascicles (hematoxylin and eosin, × 100).

**FIGURE 5:**
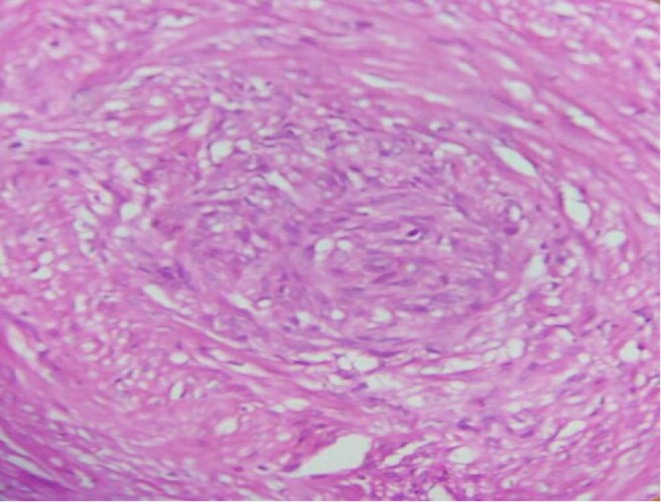
Histopathology picture shows spindle cells with cigar‐shaped nuclei (hematoxylin and eosin, × 200).

Grossly, one membranous fibro fatty tissue with multiple gray‐white solid nodules measuring 10 × 5 cm was received (Figure 2). Histopathologic examination revealed multiple well‐circumscribed mesenchymal nodules comprising bland spindle cells with cigar‐shaped nuclei and elongated eosinophilic cytoplasm forming fascicles and whorls, surrounded by mature adipose tissue (Figures 3, 4–5). There were no granulomas or necrosis seen. Consequently, a diagnosis of disseminated peritoneal leiomyomatosis was made, and TB was ruled out.

## 4 Conclusions and Results (Outcome and Follow‐Up)

The abdomen pelvic ultrasound done on the second postoperative day concluded uterine subinvolution with no mass lesion. She had a smooth postoperative period. She has been followed clinically and with ultrasound at the 3rd month, 6th month, and 1 year post‐op since MRI is not available in our setup, and she has not had any new complaints or imaging findings. She was advised about the risk of recurrence and to avoid hormonal contraceptives.

## 5 Discussion

DPL is a rare pathology. Its etiology and pathogenesis are not yet well understood. Two theories have been postulated as possible etiologies for DPL: the iatrogenic theory and the hormonal theory. The iatrogenic theory states that DPL is caused by the iatrogenic spread of uterine leiomyoma due to morcellation during myomectomy by the laparoscopic method. This procedure can leave tiny fragments of the original leiomyoma, resulting in DPL [8, 9, 10]. The second theory, the hormonal theory, hypothesizes that DPL nodules may arise from the metaplastic transformation of mesenchymal stem cells into myocytes, stimulated by high levels of progesterone and estrogen [2, 8]. The source of the excess hormone can be endogenous, as seen in pregnancy and hormone‐secreting ovarian tumors, or exogenous, in the case of hormone replacement therapies and oral contraceptive use [1, 3, 8, 11].

Most cases of DPL are asymptomatic, leading to many cases going undiagnosed. A study by Li et al. on 13 patients found that the majority (53.85%) were asymptomatic [2]. The most common presentation in symptomatic patients is abdominal pain [6, 7, 10, 12]. Patients might experience urinary symptoms, including frequent urination, irritation, and pain while urinating, and gastrointestinal symptoms, including bowel obstruction or constipation when the nodules put pressure on the urinary bladder and gastrointestinal tract, respectively [13]. Due to its asymptomatic nature, DPL is usually diagnosed incidentally during CS or other exploratory laparotomies [6, 10, 14, 15]. The study by Li et al. found that DPL was diagnosed incidentally in all 13 patients [2]. In our case, DPL was found incidentally during an emergency CS for cord prolapse. The patient had neither complaints nor image findings prior to the surgery.

DPL poses considerable diagnostic difficulties. Imaging techniques, including transvaginal ultrasound and MRI, are crucial in this context. The observation of T2 hypointensity in extrauterine masses, akin to that seen in uterine fibroids, indicates DPL [16]. Imaging studies like ultrasound, MRI, and CT scan can suggest the diagnosis of DPL preoperatively and are used for follow‐up of patients [4, 13, 16, 17]. However, these studies might give inconclusive diagnoses [6, 15]. In this case, none of the preoperative ultrasounds detect the lesion.

The definitive diagnosis of DPL is made through histologic examination of the specimen [6, 10, 13, 15]. Grossly, the tumor appears as multiple hard rubbery nodules diffusely embedded in the peritoneum [4]. Histopathologic examinations of DPL show intersecting fascicles of benign smooth muscle cells without any atypia, necrosis, or mitosis [10, 15]. Ancillary tests like immunohistochemistry (IHC) can further confirm the diagnosis, showing positive IHC stains for smooth muscle markers (smooth muscle actin [SMA] and desmin) and hormonal receptors (estrogen and progesterone) [1, 4, 7, 15].

The differential diagnosis of DPL includes malignant peritoneal mesothelioma, leiomyosarcomas, gastrointestinal stromal tumors, and peritoneal carcinomatosis [6, 7, 13]. In countries like Ethiopia, where TB is prevalent, disseminated peritoneal TB should also be considered in the differential diagnosis of DPL. In this case, the clinical diagnosis was disseminated peritoneal TB, which was ruled out after histologic examination.

Surgical excision of all tumors is the preferred treatment modality [1, 3]. Drug treatments with gonadotropin‐releasing hormone (GnRH) agonists and aromatase inhibitors can be used in patients who prefer not to have surgery [10]. Most cases regress without any intervention after discontinuation of estrogen stimulation, such as pregnancy or oral contraceptives [7, 11]. In our patient, there are no signs of recurrence or progression at the one‐year follow‐up. She was advised to avoid hormonal contraceptives to minimize estrogen exposure. DPL usually has a good prognosis [4].

## 6 Conclusion

DPL is a rare pathology that mimics many benign and malignant tumors and disseminated infections like TB. Therefore, considering DPL in the differential diagnosis of disseminated intra‐abdominal lesions is crucial. Histopathologic examination plays a vital role in confirming the diagnosis of DPL.

## Author Contributions

**Seblewengel Maru Wubalem:** conceptualization, data curation, resources, supervision, visualization, writing – original draft, writing – review and editing. **Shemsu Abraham Hussien:** data curation, writing – review and editing. **Endalk Shambel Ayalew:** writing – review and editing. **Kidest Melkamu Gebeyehu:** writing – review and editing.

## Funding

The authors have nothing to report.

## Consent

Written informed consent was obtained from the patient for publication of this case report and accompanying images.

## Conflicts of Interest

The authors declare no conflicts of interest.

## Data Availability

Data sharing not applicable to this article as no datasets were generated or analysed during the current study.
